# Disruption of ETV6 leads to TWIST1-dependent progression and resistance to epidermal growth factor receptor tyrosine kinase inhibitors in prostate cancer

**DOI:** 10.1186/s12943-018-0785-1

**Published:** 2018-02-19

**Authors:** Yuan-Chin Tsai, Tao Zeng, Wassim Abou-Kheir, Hsiu-Lien Yeh, Juan Juan Yin, Yi-Chao Lee, Wei-Yu Chen, Yen-Nien Liu

**Affiliations:** 1 Graduate Institute of Cancer Biology and Drug Discovery, College of Medical Science and Technology, Taipei Medical University, 250 Wu-Hsing Street, Taipei, 11031 Taiwan; 2 Department of Urology, The People’s Hospital of Jiangxi Province, Nanchang, People’s Republic of China; 3 Department of Anatomy, Cell Biology and Physiological Sciences Faculty of Medicine, American University of Beirut, Beirut, Lebanon; 4 Institute of Information System and Applications, National Tsing Hua University, Hsinchu, Taiwan; 5 Laboratory of Genitourinary Cancer Pathogenesis, National Cancer Institute, National Institutes of Health, Bethesda, MD USA; 6 Ph.D. Program for Neural Regenerative Medicine, College of Medical Science and Technology, Taipei Medical University, Taipei, Taiwan; 7 Center for Neurotrauma and Neuroregeneration, Taipei Medical University, Taipei, Taiwan; 8 Department of Pathology, School of Medicine, College of Medicine, Taipei Medical University, Taipei, Taiwan; 9 Department of Pathology, Wan Fang Hospital, Taipei Medical University, 250 Wu-Hsing Street, Taipei, 11031 Taiwan

**Keywords:** ETV6, TWIST1, EGFR, TKI

## Abstract

**Background:**

ETS variant gene 6 (ETV6) is a putative tumor suppressor and repressed by epidermal growth factor receptor (EGFR) signaling in prostate cancer. Since EGFR antagonists seem ineffective in castration-resistant prostate cancer (CRPC), we aim to study the role of ETV6 in the development of drug resistance.

**Methods:**

Etv6 target gene was validated by ChIP and promoter reporter assays. Correlation of ETV6 and TWIST1 was analyzed in human clinical datasets and tissue samples. Migration, invasion, and metastasis assays were used to measure the cellular responses after perturbation of ETV6 -TWIST1 axis. Proliferation and tumor growth in xenograft model were performed to evaluate the drug sensitivities of EGFR-tyrosine kinase inhibitors (TKIs).

**Results:**

ETV6 inhibits TWIST1 expression and disruption of ETV6 promotes TWIST1-dependent malignant phenotypes. Importantly, ETV6 is required to the anti-proliferation effects of EGFR-TKIs, partly due to the inhibitory function of ETV6 on TWIST1. We also found that EGFR-RAS signaling is tightly controlled by ETV6, supporting its role in TKI sensitivity.

**Conclusions:**

Our study demonstrates that disruption of ETV6 contributes to EGFR-TKI resistance, which is likely due to derepression of TWIST1 and activation of EGFR-RAS signaling. Our results implicate ETV6 as a potential marker for predicting efficacy of an EGFR-targeted anticancer approach. Combination treatment of TWIST1 inhibitors could sensitize the anti-proliferation effects of EGFR-TKIs.

**Electronic supplementary material:**

The online version of this article (10.1186/s12943-018-0785-1) contains supplementary material, which is available to authorized users.

## Background

ETS variant gene 6 (*ETV6*), which belongs to the E26 transformation-specific (ETS) transcription factor family, was suggested to play a role as a tumor suppressor due to observed deletions in last stage, advanced prostate cancer [1–4]; however, its biological functions remain unclear. We demonstrated that ETV6 exhibits antitumor effects suppressing proliferation and metastatic progression, and found that epidermal growth factor receptor (EGFR) signaling inhibits ETV6 through a microRNA-mediated mechanism [5]. EGFR signaling is involved in prostate cancer progression [6–9]; however, single-agent therapy using an EGFR tyrosine kinase inhibitor (TKI) was ineffective in castration-resistant prostate cancer (CRPC) [10]. Since resistance to EGFR antagonists eventually develops and remains a challenging phenomenon [11], it is possible that loss of ETV6 function promotes the development of drug resistance in prostate cancer.

EGFR signaling follows three general steps: a ligand-monomeric EGFR interaction, dimerization (either homodimer or heterodimer) coupled with autophosphorylation through tyrosine kinase activity, and signal transduction for tumor-related properties [11]. Therefore, mutations altering either the EGFR structure or activities of downstream components (e.g., *KRAS*) are recognized as key mechanisms responsible for resistance. However, several studies showed that the epithelial-to-mesenchymal transition (EMT) also plays a critical role in drug resistance consistent with the cancer-stem-cell (CSC) hypothesis [12, 13]. Indeed, the EMT-based gene profile is a powerful predictor of resistance to EGFR inhibitors [14]. Also, one important transcription factor of the EMT, TWIST1 [15], was found to be associated with EGFR activation [16–19] and attributable to drug resistance [20–22]. In prostate cancer, Twist family BHLH transcription factor 1 (TWIST1) was found to be highly expressed in 90% of prostate cancers compared to 6.7% of benign hyperplasia [22] and is involved in the development of CRPC [23]. Consistent with the CSC hypothesis, TWIST1 promotes tumor sphere formation, a functional indication of the self-renewal ability and CSC population [21].

Both *PTEN* and *TP53* are frequently disrupted in prostate cancer; in addition, *TP53* mutations occur in half of all CRPC [24, 25]. Following our earlier studies of ETV6 [5], we continued to investigate the molecular mechanism underlying its antitumor effects by utilizing prostate cancer cells derived from a prostate-specific *Pten/Trp53* double-knockout mouse [24, 26]. We demonstrated that Etv6 associates at the promoter region of *Twist1* and suppresses its transcription in a sequence-dependent manner. In human prostate cancer cells, ETV6 also inhibits *TWIST1* expression and ETV6-knockdown can promote TWIST1-dependent malignant phenotypes. Importantly, perturbation of ETV6-TWIST1 axis can contribute to development of drug resistance. Prostate cancer cells with ETV6-knockdown are insensitive to TKIs while exogenous expression of ETV6 restores the anti-proliferative effects in the TKI-resistant RasB1 cell line, which expresses a mutated RAS oncogene [27, 28]. We also found an inhibitory circuit between ETV6 and EGFR-RAS signaling; therefore, there could be multiple mechanisms accounting for the drug-sensitizing effect of ETV6. Our results provide a molecular mechanism by which ETV6 suppresses tumor progression through transcriptional regulation of TWIST1 and disruption of EGFR-RAS signaling.

## Methods

### Cells, constructs, and reagents

The mouse AC1, AC3, C1, and C2 cell lines were isolated from PbCre4^+^;*Pten*^*fl/fl*^*;TP53*^*fl/fl*^ Luc + mouse prostate tumors and were established as previously described [24, 26]. AC1 and AC3 cells were cultured in PrEGM medium (Lonza, Walkersville, MD, USA); C1 cells were cultured in PrEGM/DHT with 5% serum and 5% 3 T3-conditioned medium; C2 cells were cultured in PrEGM/DHT with 5% 3 T3-conditioned medium. The mouse wild-type (WT) prostatic basal cell line was provided by Dr. Lei Fang (NCI/NIH, Bethesda, MD, USA) and was cultured in WIT-P medium (Stemgent, San Diego, CA, USA) as previously described. DU145, PC3, LNCaP, and 22RV1 human prostate cancer cell lines were obtained from ATCC (Rockville, MD, USA). The metastatic RasB1 cell line was previously characterized and used to study molecular mechanisms of prostate cancer metastasis in multiple peer-reviewed articles [27–33]. All human prostate cancer cell lines were cultured in RPMI 1640 medium supplemented with 10% fetal bovine serum (FBS). RasB1 and PC3 cells with stable expression of ETV6 were established by transfection with an ETV6 complementary (c)DNA-encoding or empty pCDH-CMV-MCS-EF1-Puro vector (System Biosciences, Palo Alto, CA, USA); 2 × 10^5^ cells were seeded and transfected with 5 μg DNA and selected with puromycin for 1 month. Mouse and human ON-TARGETplus SMARTpool siRNAs (scrambled and ETV6) and a human shRNA vector (LacZ and ETV6) were from Dharmacon (Thermo Scientific, Waltham, MA, USA) and the RNAi Core Lab (Academia Sinica, Taipei, Taiwan), respectively. Transient transfections of plasmids and siRNAs were carried out using the X-tremeGENE HP DNA transfection reagent (Roche, CA, USA) or Lipofectamine RNAiMAX (Invitrogen, Carlsbad, CA, USA). Cells were treated with EGFR inhibitors, CI1033 (10 ng/ml) and AG1478 (10 μM) for 24 h in medium containing 10% serum. For EGF treatment, cells were subjected to serum-starvation for 24 h, followed by the addition of 100 ng/ml EGF for 24 h also in serum-free medium. The EGF was from R&D Systems (Minneapolis, MN, USA), and the EGFR inhibitors (CI1033 and AG1478) were from Selleck (Houston, TX, USA). The mouse Etv6-binding site was located upstream of mouse *Twist1* on chromosome 12: 33957354 at GRCm38. The Twist1-red fluorescent protein (RFP) reporter containing the mouse *Twist1* promoter with the Etv6 response element was constructed using a Clone-it Enzyme free Lentivectors Kit (System Biosciences). ETV6 response element mutations were made using a Site-Directed Mutagenesis System kit (Invitrogen). All primers used for these constructs are listed in Additional file 1; Table S1. All constructs were verified by a DNA sequence analysis.

### Quantitative real-time reverse-transcription (qRT)-polymerase chain reaction (PCR)

An qRT-PCR was used to measure *Etv6, Cdh1, Twist1,* and *Vim* in mouse cell lines or *ETV6* and *TWIST1* expressions in human prostate cancer cell lines. Total RNA was isolated using the mirVana PARIS RNA isolation system (Thermo Scientific, Waltham, MA, USA). For RT, 3 μg of total RNA was used with the SuperScript III kit (Invitrogen). Samples containing primer pairs were mixed in SYBR green PCR master mix (Applied Biosystems, Waltham, MA, USA), and the amplification program was as follows: initial 95 °C for 10 min, followed by 40 cycles of 95 °C for 15 s and 60 °C for 1 min. All reactions were normalized to mouse *Gapdh* or human *GAPDH* expression and run in triplicate. All primers used for the PCR are listed in Additional file 1; Table S2.

### Chromatin Immunoprecipitation (ChIP) assay

ChIP assays were performed using the EZ magna ChIP A kit (Millipore, Billerica, MD, USA) with a modified protocol. For small interfering (si)RNA treatment, 10^7^ AC1 cells in 10-cm dishes were transfected with mouse scrambled or ETV6 siRNAs for 48 h. Cells were cross-linked with 1% formaldehyde in culture medium at room temperature for 15 min and then quenched with the addition of 1 ml of 10× glycine. Cells were washed twice with cold phosphate-buffered saline (PBS) containing a protease inhibitor (Roche) and centrifuged at 10^5^ rpm. Cell pellets were resuspended in 0.5 ml of cell Lysis Buffer (BioRad, Hercules, CA, USA) and incubated on ice for 15 min. Nuclei were collected by centrifugation at 10^5^ rpm and 4 °C for 10 min and resuspended in nuclear lysis buffer. Genomic DNA was sheared by a microtip during sonication (Branson Sonifier 250, Germany) following 15 cycles of a 20-s burst then 1 min of cooling on ice. This procedure resulted in DNA fragments sized approximately 100~ 300 bp. Sheared chromatin was aliquoted to perform immunoprecipitation with a control immunoglobulin G (IgG) antibody (Santa Cruz Biotechnology, Santa Cruz, CA, USA) or antibodies against ETV6 or Gapdh at 4 °C overnight. A qPCR was performed in triplicate with 2 μl of eluted chromatin. ChIP antibodies and PCR primers are listed in Additional file 1; Table S3.

### Promoter reporter assay

For promoter reporter assays, AC1 or AC3 cells in 12-well plates (5 × 10^4^ cells/well) were transiently transfected with 1 μg of the mouse *Twist1*-RFP reporter containing the Etv6 response element (RE). Cells were also pretreated with 100 nM siRNA (scrambled, mouse Etv6) or 1 μg DNA (empty vector or Etv6-expressing vector, OriGene, Rockville, MD, USA) by transfection. The promoter function was analyzed using fluorescence-activated cell sorting (FACS, BD Biosciences, San Jose, CA, USA), and relative median fluorescent intensity (MFI) values were measured as previously described [28]. The MFI value for the RFP was measured by FACS using FACSDiva software (BD Biosciences) and was normalized to the value of the vehicle. Three independent experiments were run with triplicate samples.

### Correlation analyses using human gene expression datasets

To compare ETV6 expression levels with prostate cancer progression and with TWIST1 expression levels, we used mRNA expression data from human prostate cancer databases of the Taylor dataset [3] and the Cancer Genome Atlas (TCGA). The study using the Taylor dataset was conducted under Memorial-Sloan Kettering Cancer Center (MSKCC) Institutional Review Board approval on 98 primary and 13 metastatic prostate cancer samples in addition to 28 normal prostate samples. Analysis of TCGA dataset was performed with 48 normal solid tissues.

### Western blot analysis

Cells grown on 6-well plates (10^6^ cells/well) were lysed in 150 μl RIPA buffer containing complete protease inhibitors (Roche) and phosphatase inhibitors (Roche), 25 mM β-glycerophosphate, 10 mM sodium fluoride, and 1 mM sodium vanadate. Twenty micrograms of protein was separated per lane by sodium dodecylsulfate (SDS)-gel electrophoresis. After being transferred to polyvinylidene difluoride membrane, blots were blocked with 5% BSA in PBST. Primary antibodies were incubated overnight at 4 °C, and secondary antibodies were incubated at room temperature for 1 h as indicated in Additional file 1; Table S4.

### Tissue samples

Twenty-two cases of prostatic adenocarcinoma were collected from the Taipei Medical University Joint human biological database (Taipei, Taiwan), and approved by the Taipei Medical University-Joint Institutional Review Board (approval no.: 201311034). RNA was extracted from dissected tissue containing greater than 70% tumor cell content. The method for separating the specimens into two groups of ‘low’ (TWIST1_L) and ‘high’ TWIST1 (TWIST1_H) expressions was pre-decided by half of the number of patients according to TWIST1 levels by an RT-qPCR.

### Migration and invasion assay

For the migration and invasion assay, metastatic RasB1 and PC3 cells were stably transfected with the ETV6 expression vector or an empty vector. DU145 cells were stably transfected with a LacZ or ETV6 shRNA vector. LNCaP and 22RV1 cells were transiently transfected with SMARTpool ETV6 siRNA or control scrambled siRNA. Cells were resuspended at a concentration of 2.5 × 10^5^ cells/ml in serum-free medium. Matrigel™ for the invasion assay was purchased from BD Biosciences (San Jose, CA, USA). Matrigel-coated transwell dishes were prepared by adding 200 μl of 10-fold serum-free medium-diluted Matrigel. In total, 2.5 × 10^5^ cells/well in serum-free medium was plated above the Matrigel. The lower chamber was filled with 600 μl of serum-containing medium or serum-free medium with the addition of 200 ng/μL EGF. Cells that had invaded the Matrigel-coated transwells in response to EGF after 12 h were fixed and stained with a 0.5% crystal violet fixative solution for 15 min. Invaded cells on the underside of the membrane were counted and quantified by an enzyme-linked immunosorbent assay (ELISA) reader at OD 550 nm for each replicate in triplicate. The migration assay used transwells without Matrigel, and cells were fixed and stained as described for the invasion assay.

### Animal studies

Animal work was performed in accordance with a protocol approved by the Taipei Medical University Animal Care and Use Committee (Taipei, Taiwan). For the metastasis analysis, DU145 cells harboring a LacZ or ETV6 shRNA vector were subjected to intracardiac injections into 5-week-old male nude mice (National Laboratory Animal Center, Taipei, Taiwan; six mice/group) at 10^5^ cells per mouse. For survival studies, mice were euthanized when one of the following situations applied: 10% loss of body weight, paralysis, or head tilting. Hematoxylin and eosin (H&E) staining of brain tissues of mice was performed on day 80 after the injection as previously described [30]. To analyze tumorigenesis, 5-week-old male nude mice (National Laboratory Animal Center; five mice/group) were subcutaneously injected with 10^6^ RasB1 cells harboring an empty or ETV6 expression vector in 50% Matrigel™ (BD Biosciences). Subcutaneous tumors were harvested and measured from mice after treatment with 20 mg/kg CI1033 or DMSO as the control for 1 month as previously described [28].

### Proliferation assay

DU145 cells were stably transfected with a LacZ or ETV6 or TWIST1 short hairpin (sh)RNA vector, or RasB1 cells were stably transfected with an empty or ETV6 or TWIST1 expression vector, and seeded at a density of 2 × 10^3^ cells/well in 96-well plates. Cells were treated with 0, 0.1, 0.5, 1, 2, 5, and 10 nM CI1033 or 0, 0.1, 0.5, 1, 2, 5, and 10 μM AG1478 for 24 h, and analyzed using a Cell Proliferation Assay Kit (Promega, Madison, WI, USA) according to the manufacturer’s protocol.

### Statistical analysis

All data are presented as the mean ± standard error of the mean (SEM). Statistical calculations were performed with GraphPad Prism analytical tools. Differences between individual groups were determined by Student’s *t*-test or a one-way analysis of variance (ANOVA) followed by Bonferroni’s post-test for comparisons among three or more groups. The association between ETV6 and TWIST1 expressions was compared using a Chi-squared test. *p* values of < 0.05 were considered statistically significant.

## Results

### Etv6 recognizes the promoter of Twist1 and suppresses its transcription

**Fig. 1:**
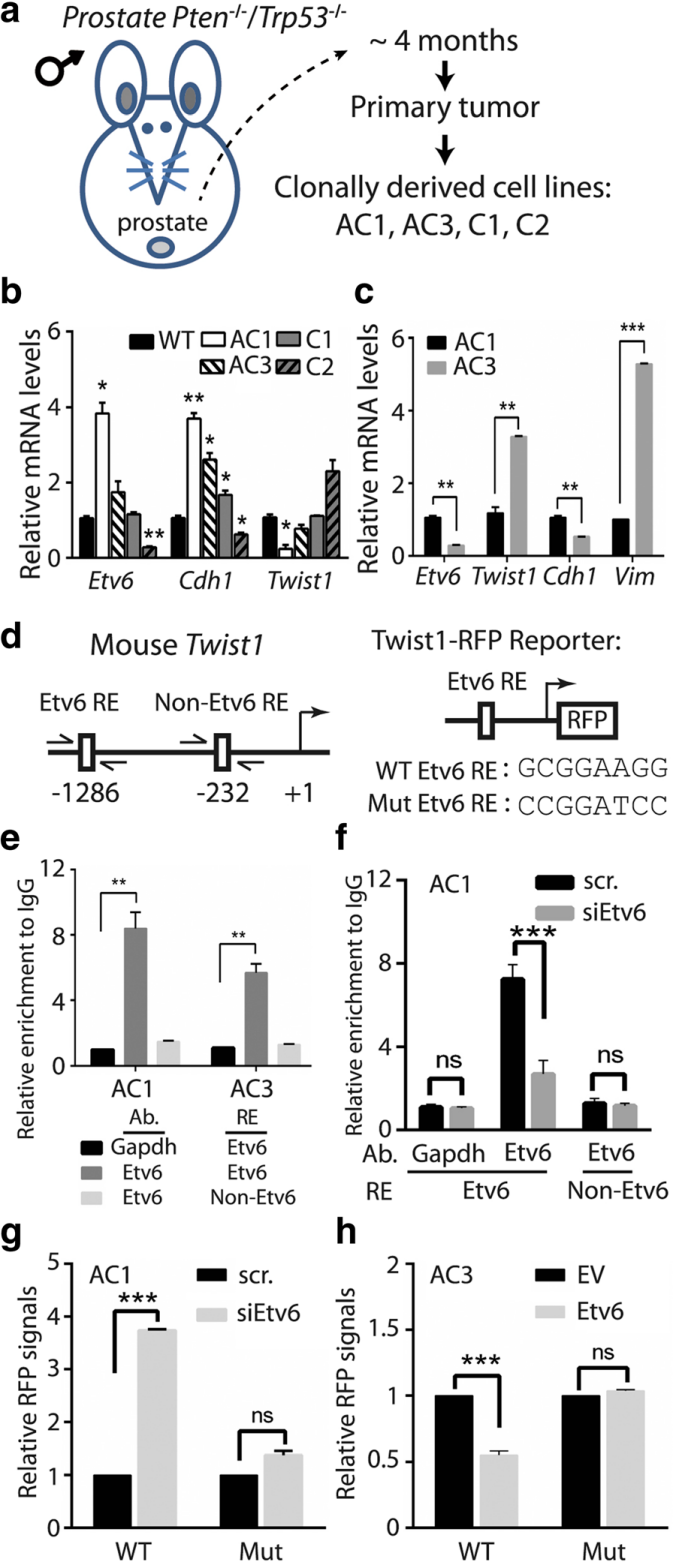
*Twist1* is transcriptionally suppressed by Etv6. (**a**) Establishment of mouse prostate cancer cell lines (AC1, AC3, C1, and C2) from primary tumors of prostate-specific *Pten/Tp53-*null mice. (**b**) Monitoring of mRNA in a wild-type (WT) mouse prostatic basal cell line and four mouse prostate cancer cell lines. (**c**) Monitoring of mRNA in AC1 and AC3 prostate cell lines. (**d**) Left: Schematic of a predicted Etv6 response element (Etv6 RE) and a non-specific site (non-Etv6 RE) on the mouse *Twist1* promoter. Right: Mouse Twist1-red fluorescent protein (RFP) reporter construct containing the WT or mutated ETV6 response element (WT vs. Mut), followed by the RFP. (**e**) ChIP analysis using an antibody (Ab) against the Etv6 protein at two RE sites (Etv6 vs. non-Etv6) in two mouse prostate cancer cell lines. An antibody against GAPDH at Etv6 RE served as a nonspecific control. The signal was determined as a percentage of the total input and was then normalized to immunoglobulin G (IgG). (**f**) ChIP analysis in response to Etv6 knockdown (siEtv6). scr., control siRNA. (**g**, **h**) Twist1-RFP reporter assay. The signal of the reporter construct containing either a WT or Mut Etv6 RE was measured in response to Etv6-knockdown (scr. vs. siEtv6, panel **g**) or Etv6 expression by transient transfection (EV vs. Etv6, panel **h**). EV, control vector. Quantification of mRNA was normalized to *Gapdh,* and results are presented as the mean ± SEM, *n* = 3. * *p* < 0.05, ** *p* < 0.01, *** *p* < 0.001; ns, non-significant

When investigating the role of ETV6 in tumor progression, we hypothesized that ETV6 was inhibitory to the EMT. Our earlier study concluded that loss of *Pten* and *TP53*, two common genetic lesions in prostate cancer, promotes the EMT and cell plasticity in a mouse prostate-specific *Pten/Trp53* double-knockout mouse model [24, 26]. When comparing the expression between one clonally derived cell line from this model and that of normal mouse prostate tissues, many EMT transcription factors were increased, while *Etv6* was comparatively reduced (Additional file 1; Figure S1A). We further investigated a panel of mouse prostate cancer cell lines derived from the mouse model (Fig. 1A), and specifically analyzed the expression pattern of *Twist1* due to its association with prostate cancer [22, 23]. Consistent with our hypothesis, *Etv6* was positively correlated with an epithelial marker (*Cdh1*) (Pearson correlation, *r* = 0.966) but negatively with *Twist1* (Pearson correlation, *r* = − 0.9002) (Fig. 1B). We further focused on a comparison between AC3 and AC1, since AC3, but not AC1, exhibits the transforming growth factor (TGF)-β-induced EMT [26]. Again, both *Etv6* and *Cdh1* were reduced, while the mesenchymal markers (*Twist1* and *Vim*) were increased in AC3 (Fig. 1C). In summary, our results showed that Etv6 is negatively associated with the EMT.

Because Etv6 is a transcription repressor [34], we searched for a consensus sequence on the promoter of *Twist1* and identified one Etv6 response element (RE) at − 1286 (Fig. 1D). We performed chromatin immunoprecipitation (ChIP) assays and found an enriched signal at the Etv6 RE only when using an antibody against Etv6, but not the antibody against Gapdh, suggesting that the signal was specific to Etv6 (Fig. 1E). When using the same Etv6-specific antibody, we observed no enrichment at a site containing no Etv6-consensus sequence (Non-Etv6 RE), suggesting that the binding was sequence-specific (Fig. 1E). In addition, the ChIP signal derived from the Etv6 RE was significantly reduced after Etv6-knockdown using Etv6-specific siRNA (siEtv6), further supporting that binding was Etv6-dependent (Fig. 1F). We thus analyzed the cis-effect of the Etv6 RE on transcription by performing a reporter assay. The reporter activity of the construct containing the WT Etv6 RE was increased after Etv6 knockdown (scr. vs. siEtv6, Fig. 1G), while it was reduced after expression of exogenous Etv6 (EV vs. Etv6, Fig. 1H). The reporter construct containing a mutated (Mut) Etv6 RE was not responsive to different ETV6 statuses (Fig. 1G, H). These results suggest that Etv6 inhibits the EMT, partly through suppression of Twist1 by a physical interaction at the promoter of *Twist1*.

### ETV6 is inversely correlated with TWIST1 in human prostate cancer

**Fig. 2:**
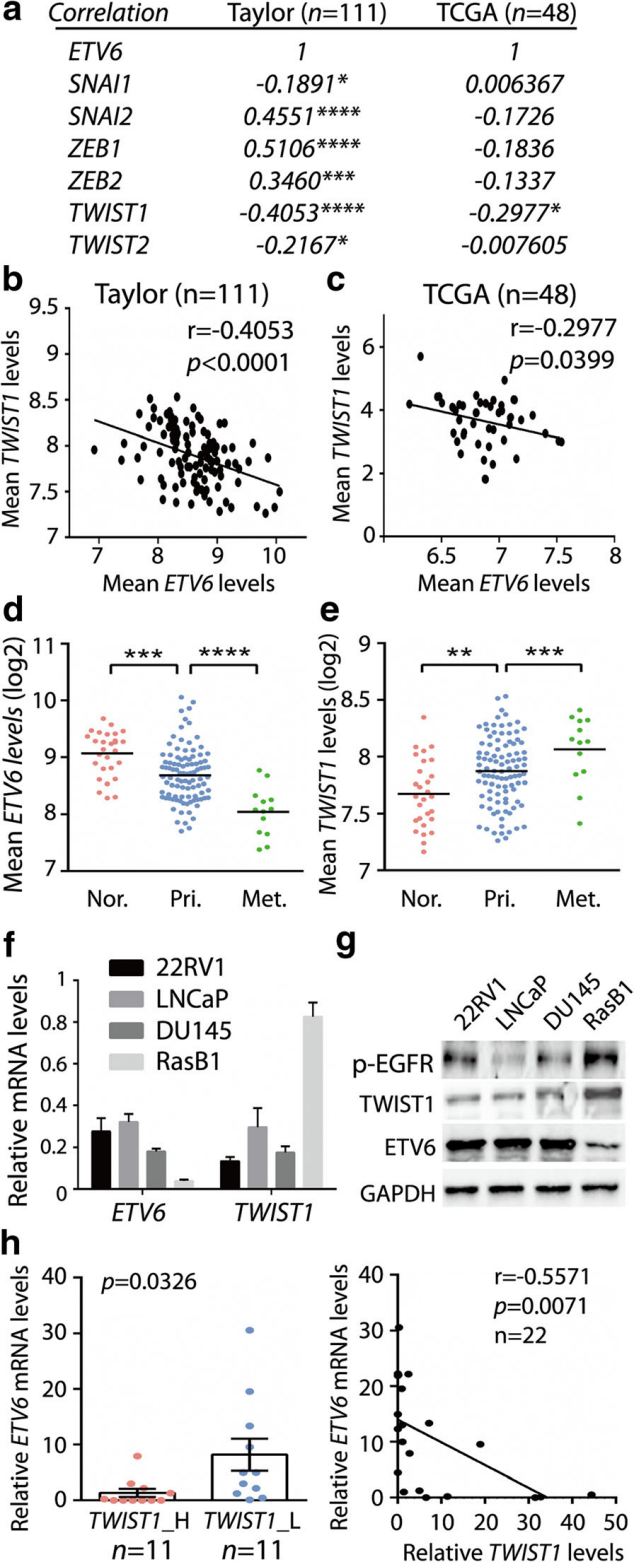
ETV6 is inversely correlated with TWIST1 in human prostate cancer. (**a**) Pearson correlation analysis between *ETV6* and epithelial-to-mesenchymal transition (EMT) transcription factors, using the Taylor and TCGA clinical prostate cancer datasets. (**b**, **c**) Correlation profiles between *TWIST1* and *ETV6* in the Taylor (**b**) and TCGA (**c**) clinical prostate cancer datasets. (**d**, **e**) Mean mRNA analysis of *ETV6* (D) or *TWIST1* (**e**) in normal prostate tissues (Nor., *n* = 28), and primary (Pri., *n* = 98) and metastatic (Met., *n* = 13) prostate cancer of the Taylor prostate cancer dataset. (**f**) Monitoring *ETV6* and *TWIST1* mRNAs in a panel of human prostate cancer cells. Gene expression was normalized to *GAPDH*. (**g**) Measurement of ETV6 and TWIST1 protein in prostate cancer cells by a Western blot assay. (**h**) Quantification of mRNA in prostate cancer tissue samples from the Taipei Medical University Joint human biological database. Left panel: Association analysis between *ETV6* and *TWIST1* in samples with two relative TWIST1 levels (H, high; L, low, *n* = 11 each group). Right panel: Pearson correlation analysis between *ETV6* and *TWIST1* mRNA in the same set of human prostate samples. Significance was determined by the Gaussian population (Pearson) and a two-tailed test. * *p* < 0.05, ** *p* < 0.01, *** *p* < 0.001, **** *p* < 0.0001

To further confirm the negative relationship between ETV6 and TWIST1 in human prostate cancer, we analyzed two public prostate cancer datasets. Many EMT drivers either showed positive or no significant correlation, instead of a negative one (Fig. 2A); however, only *TWIST1* showed a negative correlation with *ETV6* in both datasets, supporting ETV6-specific regulation (Fig. 2B, C). In addition, in the Taylor prostate dataset [3], mean expression of the *ETV6* gene was significantly lower in primary tumors and further reduced in metastatic tumors compared those in normal tissues (Fig. 2D). On the contrary, the distribution profile was completely reversed with *TWIST*1 (Fig. 2E). Consistent with the profile analyzed from the Taylor dataset, both the messenger (m)RNA and protein levels of ETV6 were lower in the metastatic RasB1 cell line than those in non-metastatic cells (22RV1, LNCaP, and DU145) (Fig. 2F, G). Again, TWIST1 was negatively associated with ETV6 and increased only in metastatic RasB1 cells (Fig. 2F, G). We further investigated the relationship between ETV6 and TWIST1 in human prostate cancer tissues collected from the Taipei Medical University Joint human biological database (approval no.: 201,311,034, Taipei, Taiwan). We divided samples into two groups based on relative TWIST1 levels (TWIST1_H vs. TWIST1_L) and found a negative association between ETV6 and TWIST1 (Fig. 2H, left panel). An inverse correlation was also demonstrated in the same set of tissue samples (Fig. 2H, right panel). In summary, we concluded that TWIST1 is negatively associated with ETV6 and is involved in tumor progression in human prostate cancer.

### ETV6 tightly controls TWIST1 expression and serves as a mediator of EGFR-TWIST1 signaling in human prostate cancer cells

**Fig. 3:**
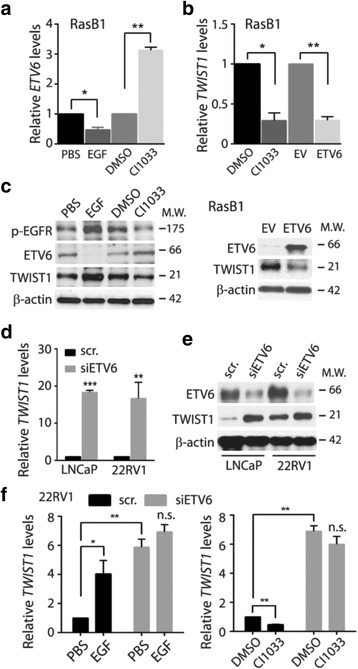
Epidermal growth factor receptor (EGFR) signaling triggers ETV6-mediated suppression of TWIST1. (**a**) Monitoring *ETV6* mRNA following treatments with EGF and CI1033. PBS and DMSO, vehicle control of EGF and CI1033, respectively. (**b**) Monitoring *TWIST1* mRNA following treatments with CI1033 and stable ETV6 expression. EV, control vector. (**c**) Western blot assay with cellular lysates from RasB1 cells. Cells were treated with EGFR modulators (EGF, CI1033, left panel) or a stable ETV6-expressing vector (EV vs. ETV6, right panel). (**d**, **e**) Two androgen receptor (AR)-positive cell lines transiently transfected with ETV6-specific siRNA (scr. vs. siETV6) were analyzed for *TWIST1* mRNA (**d**) and for the Western blot assay (**e**). (**f**) Monitoring *TWIST1* mRNA in 22RV1 cells transiently transfected with ETV6-specific siRNA (scr. vs. siETV6). *TWIST1* mRNA was measured in response to EGFR activation (PBS vs. EGF) or inactivation (DMSO vs. CI1033). Quantification of mRNA is presented as the mean ± SEM, *n* = 3. *p < 0.05, **p < 0.01, ***p < 0.001

ETV6 exhibits antitumor effects and can be negatively regulated by EGFR signaling [5]. Since TWIST1 was shown to be a downstream effector following EGFR activation [16–19], ETV6 could be a crucial component in the pathway. To test this possibility, we confirmed that EGFR activity negatively regulated *ETV6* mRNA (Fig. 3A), and that using either an EGFR inhibitor (CI1033) or overexpression of *ETV6* could reduce *TWIST1* mRNA in metastatic RasB1 cells (Fig. 3B). We observed the same effects by monitoring their protein levels (Fig. 3C). In non-metastatic prostate cancer cells, which express more ETV6 compared to RasB1 (Fig. 2G), ETV6 knockdown efficiently increased TWIST1 at both the mRNA and protein levels (Fig. 3D, E), suggesting that TWIST1 is tightly controlled by ETV6. To determine whether ETV6 is involved in EGFR-TWIST1 signaling, we found that treatments modulating EGFR activities (i.e., EGF and CI1033) no longer affected *TWIST1* following ETV6 knockdown (siETV6, Fig. 3F). Our results support a novel EGFR-ETV6-TWIST1 pathway in that ETV6 serves as a gatekeeper to maintain TWIST1 at low levels in prostate cancer.

### ETV6-knockdown leads to TWIST1-dependent malignant progression

**Fig. 4:**
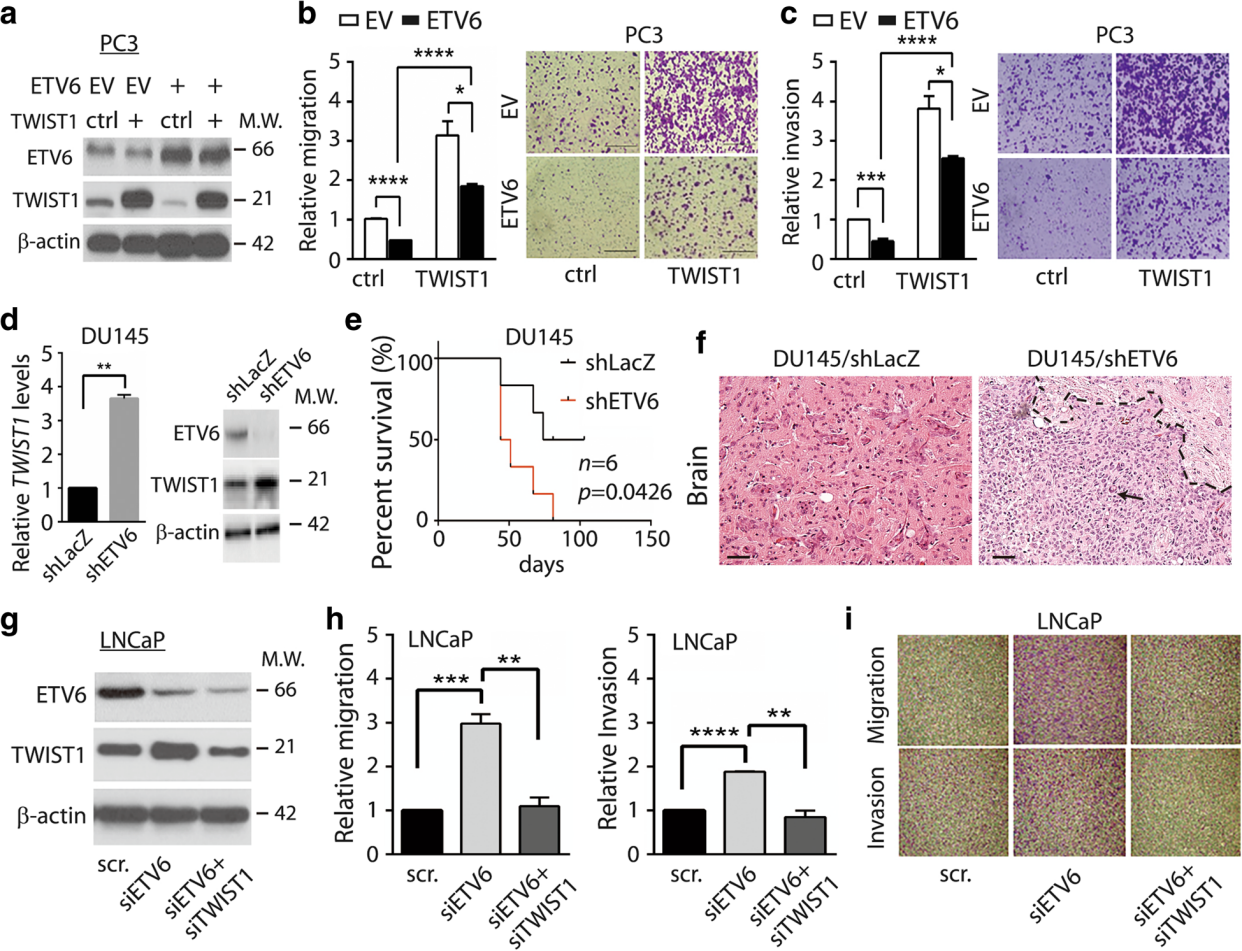
Disruption of ETV6 leads to TWIST1-dependent malignant phenotypes in prostate cancer cells. (**a**) Western blot assay of PC3 cells with stable expression of ETV6 followed by transient transfection of a TWIST1-expressing vector. EV and ctrl, control vectors of ETV6 and TWIST1, respectively. (**b**, **c**) PC3 cells with a combination of ETV6 and TWIST1 expressions as in panel **a** were analyzed by migration (**b**) or invasion (**c**) assays. Selected images are shown on the right. (**d**) Quantification of *TWIST1* mRNA and the Western blot assay in derivative cell lines of DU145. A cell line with stable ETV6-knockdown (shETV6) was generated by a lentiviral approach. shLacZ, control. (**e**) Survival analysis of mice challenged with derivatives of the DU145 cell line. (**f**) Selected images of brain tissues from mice treated with prostate cancer cells as in panel **e**. The metastatic tumor is distinguished from normal brain tissues by a dashed line and is labeled with an arrow. (**g**) Western blot assay of LNCaP cells with a combination of ETV6- and TWIST1-knockdown by specific siRNA (siETV6, siETV6 + siTWSIT1). scr, control siRNA. (**h**) Migration and invasion assays of LNCaP cells with a combination of ETV6 and TWIST1 knockdown. (**i**) Representative images of results from panel **h**. Data are presented as the mean ± SEM, *n* = 3. *p < 0.05, **p < 0.01, *** *p* < 0.001, ****p < 0.0001

We demonstrated that ETV6 efficiently suppressed metastasis of prostate cancer [5]; however, the underlying mechanism remained unclear. Based on current findings, decreasing *TWIST1* expression could account for the antitumor effects of ETV6. To test this idea, we first confirmed the metastasis-related functions of TWIST1 by monitoring a metastatic PC3 cell line stably expressing exogenous ETV6, followed by transient expression of TWIST1 (Fig. 4A). Although ETV6 did suppress the malignant abilities (both migration and invasion), overexpression of TWIST1 clearly enhanced those in the presence of ETV6 (ETV6 vs. ETV6 + TWIST1, Fig. 4B, C). We observed the same malignant effects of TWIST1 when performing these experiments in RasB1 cells (Additional file 1; Figure S1B).

Consistent with the antitumor function of ETV6, stable ETV6-knockdown in DU145 cells efficiently promoted the malignant abilities (shLacZ vs. shETV6, Additional file 1; Figure S1C). Again, we confirmed that TWIST1 was induced after ETV6-knockdown (Fig. 4D). When simulating the metastasis process by delivering this pair of cells into mice by an intracardiac injection, ETV6-knockdown indeed reduced the life expectancy (Fig. 4E) and promoted metastasis according to tumor masses detected in the brain (arrow, Fig. 4F). Since DU145 was derived from a human prostate adenocarcinoma metastasizing to the brain [35], ETV6 knockdown could reactivate the metastatic properties of DU145 to the brain. To test whether the malignant effects following ETV6-knockdown were due to increased TWIST1, we successfully reduced both ETV6 and TWIST1 by specific siRNAs in a non-metastatic LNCaP cell line (Fig. 4G). Compared to ETV6-knockdown alone, which increased migration and invasion, additional TWIST1-knockdown reduced both functions to background levels (siETV6 vs. siETV6 + siTWIST1, Fig. 4H, I). We observed the same results when performing experiments using 22RV1 cells (Additional file 1; Figure S1D-F). In summary, we concluded that ETV6-knockdown leads to derepression of TWIST1 which contributes to tumor progression.

### ETV6-TWIST1 signaling is involved in the development of resistance to EGFR antagonists

**Fig. 5:**
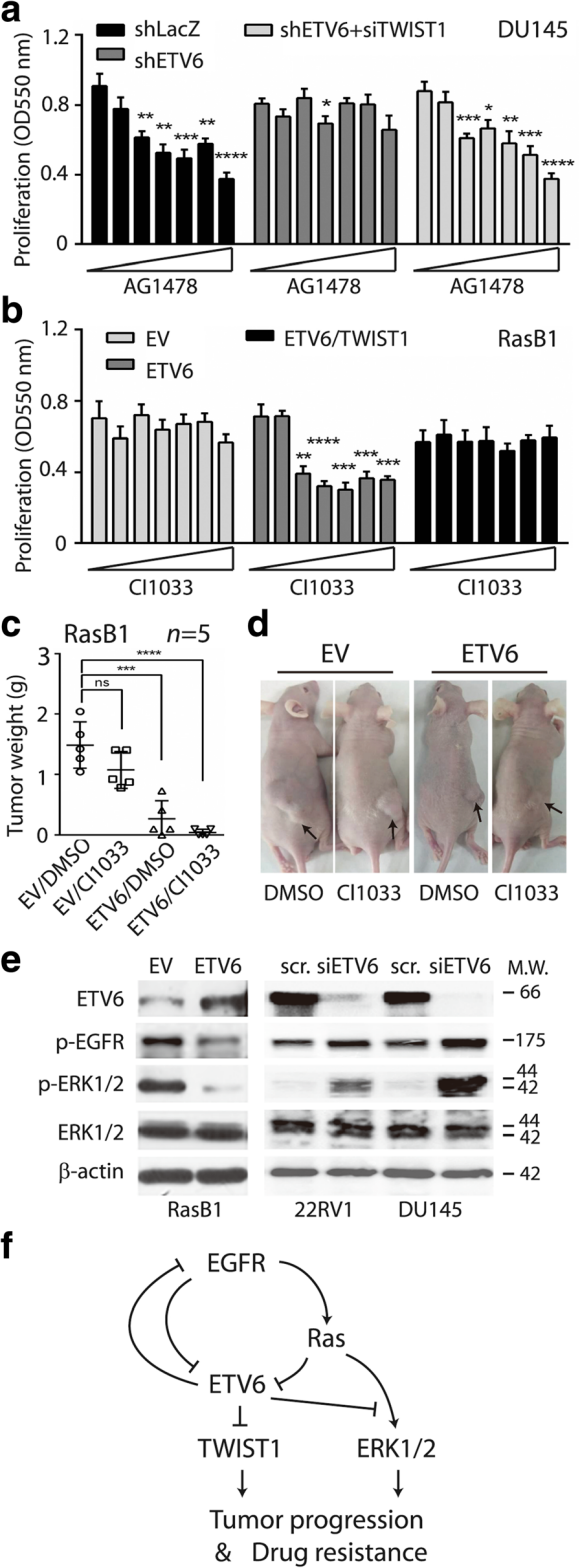
ETV6-TWIST1 signaling is involved in the molecular mechanism of drug resistance. (**a**) Proliferation assay in three DU145 derived cells treated with a tyrosine kinase inhibitor (TKI: AG1478, 0.1~ 10 μM), *n =* 8. shLacZ, control; shETV6, ETV6-knockdown; shETV6 + siTWIST1, both ETV6- and TWIST1-knockdown. (**b**) Proliferation assay in three stable RasB1 derived cells treated with a TKI (CI1033, 0.1~ 10 nM), *n* = 8. EV, control vector; ETV6, ETV6-expressing vector; ETV6 + TWIST1, both ETV6- and TWIST1-expressing vectors. (**c**) Tumor growth analysis of stable RasB1 cell lines (EV vs. ETV6) subcutaneously inoculated in male nude mice followed by treatment with CI1033. Tumor sizes were monitored weekly (left, *n* = 5). At the end, tumor weights were also measured (right, *n* = 5). (**d**) Selected images of mice from panel C, containing tumors (arrows) derived from stable RasB1 cell lines (EV vs. ETV6). (**e**) Western blot analysis of human prostate cancer cells. RasB1 cells were introduced with exogenous ETV6 or under ETV6-knockdown in 22RV1 and LNCaP cells by an siRNA approach (siETV6). EV, empty vector; scr., siRNA control. (**f**) Working model, activation of the epidermal growth factor receptor (EGFR) promotes tumor progression and drug resistance through RAS signaling and suppression of ETV6, which lead to TWIST1-dependent malignant phenotypes. A mutual inhibitory circuit exists between EGFR-RAS signaling and ETV6

The EGFR is a validated target for cancer therapy; however, resistance to EGFR inhibitors eventually evolves [11]. Since EGFR-based therapeutics showed no beneficial effects in prostate cancer [10, 36], it is important to determine whether the ETV6-TWIST1 axis plays a role in the development of drug resistance. We showed the anti-proliferative effects using one EGFR kinase inhibitor (AG1478) in DU145 cells; however, following ETV6-knockdown, cells became resistant (Fig. 5A). Interestingly, simultaneous knockdown of both ETV6 and TWIST1 recovered the anti-proliferative effect of the inhibitor (shETV6 + siTWIST1, Fig. 5A), consistent with our hypothesis that inducing TWIST1 after disruption of ETV6 contributes to malignant progression. The same experiment was performed using another EGFR inhibitor (CI1033, Additional file 1; Figure S1G).

The RasB1 cell line is derived from DU145 by introducing a mutant RAS and thus is resistant to EGFR TKIs [27, 28]. We demonstrated again that an EGFR antagonist (CI1033) did not suppress proliferation of RasB1, but overexpression of ETV6 clearly recovered the anti-proliferative function (EV vs. ETV6, Fig. 5B). However, RasB1 was resistant to CI1033 again after co-expression of both ETV6 and TWIST1, supporting the role of TWIST1 in the development of drug resistance (Fig. 5B). The same conclusion was derived using another TKI (AG1478, Additional file 1; Figure S1H). We also used a mouse xenograft model with the subcutaneous injection of RasB1 cells and tested the effect of ETV6 on CI1033. After the mice developed tumors, the mice were treated with CI1033 for 4 weeks. We found that CI1033 did not significantly reduce tumors (EV/DMSO vs. EV/CI1033); however, cells expressing ETV6 showed drastic antitumor effects (EV/DMSO vs. ETV6/DMSO, Fig. 5C), consistent with our previous findings [5]. Further CI1033 treatment in mice injected with RasB1 cells expressing exogenous ETV6 completely suppressed tumor growth in three of five mice tested (ETV6/CI1033, Fig. 5C, D).

### A mutual inhibition circuit exists between EGFR-RAS signaling and ETV6

In addition to the inhibitory role of ETV6 on TWIST1, we investigated the effect of ETV6 on EGFR-RAS signaling. We found that exogenous ETV6 negatively regulated the phosphorylation status of the EGFR (p-EGFR, Fig. 5E, left) in metastatic RasB1 cells. Interestingly, exogenous ETV6 also efficiently suppressed the phosphorylation status of extracellular signal-regulated kinase 1 and 2 (ERK1/2), a downstream signal transducer of EGFR-RAS signaling [37], while ETV6-knockdown in non-metastatic 22RV1 and DU145 cells increased the phosphorylation signal (p-ERK1/2, Fig. 5E, right). Thus, we hypothesized a mutual inhibition between EGFR-RAS signaling and ETV6. The inhibitory effect of ETV6 on EGFR-RAS signaling could also explain an earlier study showing that ETV6 can suppress RAS-induced transformation in an NIH3T3 cell model [38]. Based on our aggregated results, we propose a working model that disruption of ETV6 contributes to tumor progression and TKI-resistance through derepression of TWIST1 and activation of EGFR-RAS signaling (Fig. 5F).

## Discussion

An earlier study showed that the genomic locus containing *ETV6* is the most common translocation site in leukemia [39]. While one allele can fuse to over 30 different genomic loci, the other usually undergoes deletion, which accounts for the frequently observed loss of heterozygosity [38]. Therefore, *ETV6* was considered a tumor suppressor, even in certain types of solid tumors [40]. In prostate cancer, many genes from the ETS family participate in fusion transcripts with transmembrane serine protease isoform 2 (*TMPRSS2*); in fact, three ETS members (*ERG*, *ETV1*, and *ETV4*) contribute to about 80% of *TMPRSS2* fusion [41, 42]. However, *ETV6* has not yet been reported to be involved in chromosome translocation or fusion with *TMPRSS2*; instead, frequent deletions were observed in late stages, metastatic prostate cancer [1–4]. It was shown that following androgen signaling, both the androgen receptor (*AR*) and topoisomerase II beta (*TOP2B*) were localized to *TMPRSS2-ERG* genomic breakpoints, followed by TOP2B-mediated DNA breakage and recombination [43]. Therefore, genomic rearrangements in prostate cancer, although frequently occurring, are likely dependent on the nature of the AR and TOP2B and are restricted to certain ETS loci except for *ETV6*. Since genomic lesions of *ETV6* are common in leukemia, which does not rely on androgen, deletion of *ETV6* might not be dependent on AR signaling.

Androgen deprivation therapy (ADT) is a standard procedure in prostate cancer; however, patients eventually develop metastatic castration-resistant prostate cancer. Therefore, patients could benefit from combining therapeutic approaches with different mechanisms. One would consider EGFR-targeted therapy since that the majority of prostate cancers are derived from an epithelial origin and are associated with elevated activities of the EGFR family [6–9]; however, using either an EGFR antagonist or anti-HER2 antibody did not achieve therapeutic effectiveness in CRPC [10, 36]. This might reflect the challenging issue for EGFR-targeted therapy in general since resistance inevitably occurs even though the antagonists are used in many types of cancer [11]. Our proposed model that disruption of ETV6 leads to TKI resistance via derepression of both TWIST1 and EGFR-RAS signaling (Fig. 5F), provides one explanation to this issue. Since *ETV6* is frequently deleted in late stage, malignant prostate cancer [1–4], the majority of CRPC could be lack of ETV6 function, consequently, irresponsive to TKIs. Prostate cancer still at earlier or hormone-sensitive stages containing intact ETV6 activity could be responsive to EGFR-targeted antagonists as monotherapy. Patients might benefit from prescreening their ETV6 statuses (genetics or expression levels) before treated with EGFR-TKIs.

Our results support a model that the EGFR facilitates tumor malignancy by reducing ETV6, which enhances TWIST1 activities. In addition, we previously reported that activation of EGFR signaling facilitates bone metastasis of prostate cancer through EGFR-mediated transcriptional suppression of microRNA-1 (miR-1) [16]. Reduced miR-1 can increase TWIST1 function since miR-1 targets the 3′ untranslated region of *TWIST1* and destabilizes its mRNA [16]. Furthermore, an earlier study also showed that EGFR activation can trigger signal transducer and activator of transcription 3 (STAT3)-dependent transcription of *TWIST1* and EMT [17]. Based on conclusions from our studies and others, TWIST1 appears to be a key mediator that promotes malignant phenotypes; therefore, EGFR signaling utilizes multiple mechanisms to increase TWIST1 expression, including suppression of its negative regulators (miR-1 and ETV6) and activation of its positive regulator (e.g. STAT3) following EGFR activation. Therefore, designing novel TWIST1 inhibitors could sensitize the anti-proliferation effects and reduce the risk of resistance when choosing EGFR-TKIs.

## Conclusions

Our studies provide a novel and testable hypothesis that connects ETV6-TWIST1 signaling to EGFR-TKI resistance during prostate cancer progression. Information about the genetic or expressional statuses of ETV6 could be valuable for decision making in personalized medicine when considering EGFR-based therapeutics. EGFR antagonists could achieve better and more-sustainable antitumor responses in combination with TWIST1-targeted agents.

## Additional file

Additional file 1: Figure Legends and Tables. (ZIP 1350 kb)

## Abbreviations

ADT: androgen deprivation therapy
CRPC: castration-resistant prostate cancer
EGFR: epidermal growth factor receptor
EMT: epithelial-to-mesenchymal transition
ETS: E26 transformation-specific
ETV6: ETS variant gene 6
miR-1: microRNA-1
STAT3: signal transducer and activator of transcription 3
TGFβ: transforming growth factor β
TKI: tyrosine kinase inhibitor
TWIST1: Twist family BHLH transcription factor 1
