# Supplementary material for: Disruption of ETV6 leads to TWIST1-dependent progression and resistance to epidermal growth factor receptor tyrosine kinase inhibitors in prostate cancer
Source: Mol Cancer. 2018 Feb 19;17:42. doi: 10.1186/s12943-018-0785-1 (PMC5817720; doi:10.1186/s12943-018-0785-1)
Supplement: Supplementary file 1 — Figure Legends and Tables. (ZIP 1350 kb) [file 12943_2018_785_MOESM1_ESM.zip › Supplementary Information.docx]

**Disruption of ETV6 leads to TWIST1-dependent progression and resistance to epidermal growth factor receptor tyrosine kinase inhibitors in prostate cancer**

Yuan-Chin Tsai, Tao Zeng, Wassim Abou-Kheir, Hsiu-Lien Yeh, Juan Juan Yin, Yi-Chao Lee, Wei-Yu Chen, Yen-Nien Liu

**Additional file 1 figure legends**

**Additional file 1; Fig. S1. Disruption of ETV6 leads to TWIST1-dependent malignant phenotypes in prostate cancer cells.** (A) Monitoring mRNA ratios of epithelial-to-mesenchymal transition (EMT) transcription factors between C2 tumor and mouse normal prostate tissues. (B) Migration and invasion assays of RasB1 stable cell lines. EV, control vector; ETV6, ETV6-expressing vector. (C) Migration and invasion assays of DU145 stable cell lines. shLacZ, control; shETV6, ETV6 knockdown. Selective images are shown on the right. (D) Western blot assay of 22RV1 cells with a combination of ETV6- and TWIST1-knockdown by specific siRNA (siETV6, siETV6+siTWSIT1). scr, control siRNA. (E) Migration and invasion assays of 22RV1 cells with a combination of ETV6- and TWIST1-knockdown. (F) Representative images of results from panel E. (G) Proliferation assay in three DU145 stable cell lines treated with another tyrosine kinase inhibitor (CI1033, 0.1~10 nM), *n*=8. shLacZ, control; shETV6, ETV6 knockdown; shETV6+siTWIST1, both ETV6- and TWIST1-knockdown. (H) Proliferation assay in RasB1 stable cell lines treated with an AG1478 tyrosine kinase inhibitor, *n*=8. EV, control vector. ETV6, ETV6-expressing vector. Data are presented as the mean ± SEM. * *p*<0.05, ** *p*<0.01, *** *p*<0.001, **** *p* 0.0001.

**Additional file 1 tables**

**Table S1:** Primer sequences of the promoter reporter constructs

| Mouse Twist1 promoter Etv6-binding element reporter constructs | |
| --- | --- |
| Wild-type *Twist1*_Etv6RE F | CTGGCGCGGAAGGAAATC |
| Wild-type *Twist1*_Etv6RE R | TGCGAACCATTCAAAACCGA |
| Mutant *Twist1*_Etv6RE F | GCGCTCTGGCGCGGATCCAAATCGCCCCGCG |
| Mutant *Twist1*_Etv6RE R | TCCGCGCCAGAGCGCGCCCAGCGCCCCCGC |

**Table S2.** Primer sequences for the RT-qPCR

| Gene | 5'-3' |
| --- | --- |
| Mouse Twist1 F | CGGACAAGCTGAGCAAGAT |
| Mouse Twist1 R | GGACCTGGTACAGGAAGTCG |
| Mouse Etv6 F | TGCCCATTGGGAGAATAGCA |
| Mouse Etv6 R | TCCGTTGGGATCCACTATCC |
| Mouse Cdh1 F | GAC AACGCTCCTGTCTTCAA |
| Mouse Cdh1 R | ACGGTGTACACAGCTTTCCA |
| Mouse Vim F | GATGCGTGAGATGGAAGAGA |
| Mouse Vim R | GGCCATGTTAACATTGAGCA |
| Mouse Gapdh F | CAGAACATCATCCCTGCATC |
| Mouse Gapdh R | CTGCTTCACCACCTTCTTGA |
| Human TWIST1 F | CGGACAAGCTGAGCAAGAT |
| Human TWIST1 R | CTGGAGGACCTGGTAGAGGA |
| Human ETV6 F | ATAACCCTCCCACCATTGAA |
| Human ETV6 R | CGGTGATTTGTCGTGATAGG |
| Human GAPDH F | CCAGTAGAGGCAGGGATGAT |
| Human GAPDH R | CTTTCATTGTCTTTTCCGCC |

**Table S3.** Antibody information and primer sequences for the ChIP assay

| ChIP antibodies | | | | |
| --- | --- | --- | --- | --- |
| Primary antibody | Species | Clonality | Source | Dilution |
| GAPDH | Rabbit | Polyclonal | Genetex (GTX100118) | 1/50 |
| ETV6/TEL (H-214) | Rabbit | Polyclonal | Santa Cruz (sc-11382) | 1/50 |
| Immunoglobulin G | Rabbit |  | Santa Cruz (sc-2027) | 1/50 |
| ChIP primers | | | | |
| Site | 5'-3' | | | |
| mTwist1 Etv6-RE F | CTGGCGCGGAAGGAAATC | | | |
| mTwist1 Etv6-RE R | TGCGAACCATTCAAAACCGA | | | |
| Non-mTwist1 Etv6-RE F | TCGGACAAGCTGAGCAAGAT | | | |
| Non-mTwist1 Etv6-RE R | TGGGCCACATAGCTGCAG | | | |

**Table S4.** Antibody information for the Western blot analysis

| Primary antibody | Clonality | Source | Dilution | Secondary antibody | Dilution |
| --- | --- | --- | --- | --- | --- |
| p-EGFR (Tyr1068) | Monoclonal | Cell Signaling (#3777) | 1/1000 | anti-rabbit IgG (Jackson Labs) | 1/5000 |
| p-ERK1/2 (Thr202/Tyr204) | Monoclonal | Cell Signaling (#4376) | 1/1000 | anti-rabbit IgG (Jackson Labs) | 1/5000 |
| ERK1/2 | Polyclonal | Cell Signaling (#9102) | 1/1000 | anti-rabbit IgG (Jackson Labs) | 1/5000 |
| TWIST1 | Polyclonal | GeneTex (GTX127310) | 1/1000 | anti-rabbit IgG (Jackson Labs) | 1/5000 |
| ETV6 | Polyclonal | Proteintech (13182-1-AP) | 1/1000 | anti-rabbit IgG (Jackson Labs) | 1/5000 |
| GAPDH | Polyclonal | Genetex (GTX100118) | 1/1000 | anti-rabbit IgG (Jackson Labs) | 1/100000 |
| β-actin | Polyclonal | GeneTex (GTX109639) | 1/1000 | anti-rabbit IgG (Jackson Labs) | 1/20000 |
