# Supplementary figures and images for: Disruption of ETV6 leads to TWIST1-dependent progression and resistance to epidermal growth factor receptor tyrosine kinase inhibitors in prostate cancer
Source: Mol Cancer. 2018 Feb 19;17:42. doi: 10.1186/s12943-018-0785-1 (PMC5817720; doi:10.1186/s12943-018-0785-1)

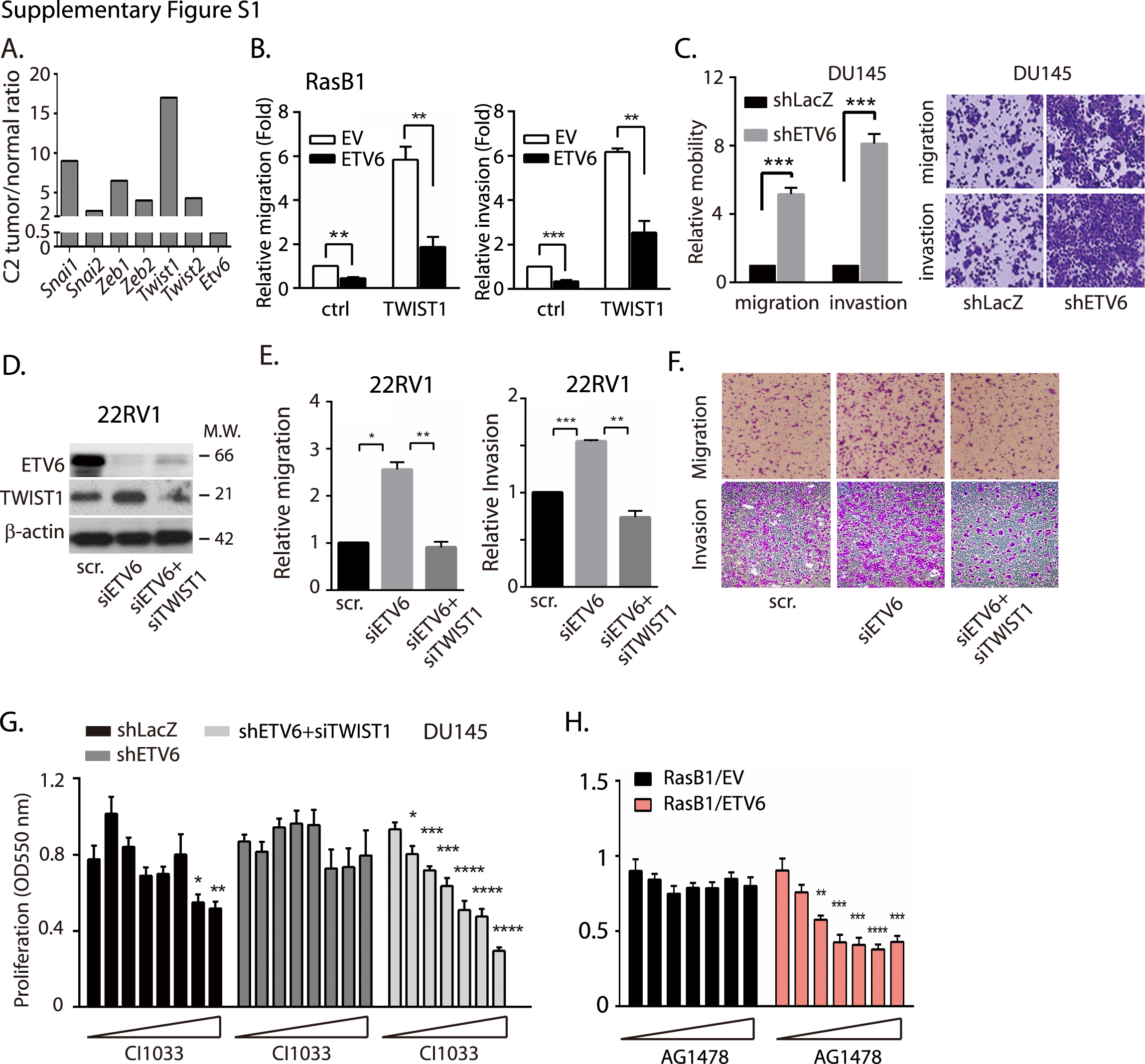

Supplement: Supplementary file 1 — Figure Legends and Tables. (ZIP 1350 kb) [file 12943_2018_785_MOESM1_ESM.zip › Sup FIG1.jpg]
